# The 2022 *Nucleic Acids Research* database issue and the online molecular biology database collection

**DOI:** 10.1093/nar/gkab1195

**Published:** 2021-12-27

**Authors:** Daniel J Rigden, Xosé M Fernández

**Affiliations:** Institute of Systems, Molecular and Integrative Biology, University of Liverpool, Crown Street, Liverpool L69 7ZB, UK; Institut Curie, 25 rue d’Ulm, 75005 Paris, France

## Abstract

The 2022 *Nucleic Acids Research* Database Issue contains 185 papers, including 87 papers reporting on new databases and 85 updates from resources previously published in the Issue. Thirteen additional manuscripts provide updates on databases most recently published elsewhere. Seven new databases focus specifically on COVID-19 and SARS-CoV-2, including SCoV2-MD, the first of the Issue's Breakthrough Articles. Major nucleic acid databases reporting updates include MODOMICS, JASPAR and miRTarBase. The AlphaFold Protein Structure Database, described in the second Breakthrough Article, is the stand-out in the protein section, where the Human Proteoform Atlas and GproteinDb are other notable new arrivals. Updates from DisProt, FuzDB and ELM comprehensively cover disordered proteins. Under the metabolism and signalling section Reactome, ConsensusPathDB, HMDB and CAZy are major returning resources. In microbial and viral genomes taxonomy and systematics are well covered by LPSN, TYGS and GTDB. Genomics resources include Ensembl, Ensembl Genomes and UCSC Genome Browser. Major returning pharmacology resource names include the IUPHAR/BPS guide and the Therapeutic Target Database. New plant databases include PlantGSAD for gene lists and qPTMplants for post-translational modifications. The entire Database Issue is freely available online on the Nucleic Acids Research website (https://academic.oup.com/nar). Our latest update to the NAR online Molecular Biology Database Collection brings the total number of entries to 1645. Following last year's major cleanup, we have updated 317 entries, listing 89 new resources and trimming 80 discontinued URLs. The current release is available at http://www.oxfordjournals.org/nar/database/c/.

## NEW AND UPDATED DATABASES

The 29th annual *Nucleic Acids Research* Database Issue contains 185 papers covering topics from across biology and beyond. The ongoing COVID-19 pandemic continues to play a major role, inspiring the construction of seven new databases (Table 1). The reader will also find its impact obvious in papers describing other new and returning databases throughout the Issue. A further 80 papers (Table 2) report on other new databases while returning databases contribute a further 85 papers. Finally, there are 13 papers from resources most recently published elsewhere (Table 3).

**Table 1.** Descriptions of new databases related to COVID-19 in the 2022 *NAR* Database issue

Database Name	URL	Short description
COVID19db	http://www.biomedical-web.com/covid19db or http://hpcc.siat.ac.cn/covid19db	SARS-CoV-2 transcriptomics and drug discovery
Ensembl COVID-19 resource	https://covid-19.ensembl.org	Integrated public SARS-CoV-2 data
ESC	http://clingen.igib.res.in/esc	SARS-CoV-2 immune escape variants
SCoV2-MD	http://www.scov2-md.org	Molecular dynamics of SARS-CoV-2 proteins and variant interpretation
SCovid	http://bio-annotation.cn/scovid	Single cell transcriptomics of SARS-CoV-2 infection
T-cell COVID-19 Atlas	https://t-cov.hse.ru	Predicted affinities between SARS-CoV-2 peptides and HLA alleles
VarEPS	https://nmdc.cn/ncovn	SARS-CoV-2 variants, known and theoretical, versus therapies

**Table 2.** Descriptions of new databases in the 2022 *NAR* Database issue not specifically related to COVID-19

Database name	URL	Short description
3′aQTL-atlas	https://wlcb.oit.uci.edu/3aQTLatlas	3′UTR alternative polyadenylation quantitative trait loci
AlphaFold Protein Structure Database	https://alphafold.ebi.ac.uk	Protein structures predicted by AlphaFold
Animal-eRNAdb	http://gong_lab.hzau.edu.cn/Animal-eRNAdb	Animal enhancer RNAs
AMDB	http://leb.snu.ac.kr/amdb	Animal Microbiome Database
ASMdb	http://www.dna-asmdb.com	Allele-Specific DNA Methylation Database
ARTS-DB	https://arts-db.ziemertlab.com	Database for Antibiotic Resistant Targets
BrainBase	https://ngdc.cncb.ac.cn/brainbase	Brain disease knowledgebase
CancerMIRNome	http://bioinfo.jialab-ucr.org/CancerMIRNome	miRNA profiles in cancer
CancerSCEM	https://ngdc.cncb.ac.cn/cancerscem	Human cancer single-cell gene expression
CeDR	https://ngdc.cncb.ac.cn/cedr	Drug responses in health and disease from scRNA-seq
CircleBase	http://circlebase.maolab.org	Human extrachromosomal circular DNA
circMine	http://www.biomedical-web.com/circmine or http://hpcc.siat.ac.cn/circmine	Human circRNA transcriptome in health and disease
CompoDynamics	https://ngdc.cncb.ac.cn/compodynamics	Sequence composition and characteristics across genomes
ConVarT	https://convart.org	Orthologous variants between human, mouse and worm
CovPDB	http://www.pharmbioinf.uni-freiburg.de/covpdb	Covalent inhibitors and their complexes
CTR-DB	http://ctrdb.ncpsb.org.cn	Patient-derived clinical transcriptomes and drug responses
CyanoOmicsDB	http://www.cyanoomics.cn	Cyanobacteria genomics and transcriptomics
DDinter	http://ddinter.scbdd.com	Drug-drug interactions
DISCO	https://www.immunesinglecell.org	Deep Integration of Single-Cell Omics
dNTPpoolDB	https://dntppool.org	dNTP concentrations *in vivo*
EVA	https://www.ebi.ac.uk/eva	European Variation Archive
EWAS Open Platform	https://ngdc.cncb.ac.cn/ewas	Analysis platform for EWAS research
Gene Expression Nebulas	https://ngdc.cncb.ac.cn/gen	Expression profiles across species, bulk and single cell
GPEdit	https://hanlab.uth.edu/GPEdit	A-to-I RNA editing in cancer
GproteinDb	https://gproteindb.org	G proteins and their interactions
GRAND	https://grand.networkmedicine.org	Human gene regulation models
gutMGene	http://bio-annotation.cn/gutmgene	Target genes of gut microbes and microbial metabolites in human and mouse
huARdb	https://huarc.net/database	Human Antigen Receptor database
Human Proteoform Atlas	http://human-proteoform-atlas.org	Human proteoforms
INDI	http://research.naturalantibody.com/nanobodies	Integrated Nanobody Database for Immunoinformatics
qPTMplants	http://qptmplants.omicsbio.info	Plant PTMs, including quantitation
Kincore	http://dunbrack3.fccc.edu/kincore	Protein kinase sequence, structure and phylogeny
LIRBase	https://venyao.xyz/lirbase	Long Inverted Repeats in eukaryotes
lncRNAfunc	https://ccsm.uth.edu/lncRNAfunc	Regulatory roles of lncRNAs in cancer
m5C-Atlas	http://www.xjtlu.edu.cn/biologicalsciences/m5c-atlas	The 5-methylcytosine (m5C) epitranscriptome
mBodyMap	https://mbodymap.microbiome.cloud/#/mbodymap	Distribution of microbes across the human body in health and disease
MetazExp	http://bioinfo.njau.edu.cn/metaExp	Analysis of gene expression and alternative splicing in metazoans
miTED	http://microrna.gr/mited	microRNA Tissue Expression Database
msRepDB	https://msrepdb.cbrc.kaust.edu.sa/pages/msRepDB/index.html	multi-species Repeat DataBase
MVIP	https://mvip.whu.edu.cn	Multi-omics Portal of Viral Infection
Nanobase.org	https://nanobase.org	DNA, RNA or protein-DNA/RNA hybrid nanostructures
NCATS Inxight: Drugs	https://drugs.ncats.io	Drugs, their properties and regulation
NMDC Data Portal	https://data.microbiomedata.org	Multi-omics microbiome data
NPCDR	https://idrblab.org/npcdr or http://npcdr.idrblab.net	Drug-Natural Product combinations and diseases
NP-MRD	http://np-mrd.org	Natural Products Magnetic Resonance Database
OlfactionBase	https://olfab.iiita.ac.in/olfactionbase	Odors, Odorants and Olfactory Receptors
OncoDB	http://www.oncodb.org.	Gene Expression and Viral Infection in Cancer
ONQUADRO	http://onquadro.cs.put.poznan.pl	DNA and RNA quadruplexes
PCMDB	http://www.tobaccodb.org/pcmdb	Plant Cell Marker Database
PlantGSAD	http://systemsbiology.cau.edu.cn/PlantGSEAv2	Plant gene set annotations
PncsHub	https://pncshub.erc.monash.edu	Non-classically secreted proteins in Gram-positive bacteria
Pol3Base	http://rna.sysu.edu.cn/pol3base/index.php	PolIII-transcribed ncRNAs
proCHiPdb	http://prochipdb.org	Chromatin immunoprecipitation database for prokaryotic organisms
PopHumanVar	https://pophumanvar.uab.cat	Causal variants of selective sweeps
ProNAB	https://web.iitm.ac.in/bioinfo2/pronab	Protein-Nucleic Acid Binding affinity
Regeneration Roadmap	https://ngdc.cncb.ac.cn/regeneration/index	Literature and multi-omics data on cell regeneration
R-loopBase	https://rloopbase.nju.edu.cn	R-loops and R-loop regulators
RNAPhaSep	http://www.rnaphasep.cn	RNAs involved in liquid-liquid phase separation
RPS	http://rps.renlab.org	RNAs involved in liquid-liquid phase separation
scAPAatlas	http://www.bioailab.com:3838/scAPAatlas	scRNAseq-based analysis of alternative polyadenylation across cells, tissues and species
scAPAdb	http://www.bmibig.cn/scAPAdb	scRNAseq-based analysis of alternative polyadenylation across cells, tissues and species
scEnhancer	http://enhanceratlas.net/scenhancer	Single-cell enhancer resource
scMethBank	https://ngdc.cncb.ac.cn/methbank/scm	Single Cell methylation data
SomaMutDB	https://vijglab.einsteinmed.org/SomaMutDB	Somatic mutations in normal human tissues
SPENCER	http://spencer.renlab.org	Cancer-associated ncRNA-encoded small peptides
SPICA	https://spica.unil.ch	Swiss Portal for Immune Cell Analysis
SYNBIP	https://idrblab.org/synbip	Synthetic binding proteins
TcoFBase	http://bio.liclab.net/TcoFbase	Transcription cofactors in human and mouse
TF-Marker	http://bio.liclab.net/TF-Marker	Human transcription factors, especially as cell markers
TISMO	http://tismo.cistrome.org	Mouse syngeneic tumor models
TissueNexus	https://www.diseaselinks.com/TissueNexus	Tissue or cell line functional gene networks
TransLnc	http://bio-bigdata.hrbmu.edu.cn/TransLnc	Coding potential of lncRNAs across tissues, including neoantigens
tsRFun	http://biomed.nscc-gz.cn/DB/tsRFun	tsRNA expression and networks
VannoPortal	http://mulinlab.org/vportal	Human genetic variants vs traits and diseases
VEuPathDB	http://VEuPathDB.org	Eukaryotic pathogens, their vectors and hosts
ViMIC	http://bmtongji.cn/ViMIC/index.php	Virus Mutations, Integration sites and Cis-effects
ViroidDB	https://viroids.org	Viroids and viroid-like circular RNA agents
VThunter	https://db.cngb.org/VThunter	scRNA-seq-based analysis of virus receptor expression across animals
webTWAS	http://www.webtwas.net	Transcriptome-Wide Association Studies
ZOVER	http://www.mgc.ac.cn/cgi-bin/ZOVER/main.cgi	Zoonotic and vector-borne viruses

**Table 3.** Updated descriptions of databases most recently published elsewhere

Database name	URL	Short description
BRAD	http://brassicadb.cn	Brassica Database
CPLM	http://cplm.biocuckoo.cn	Compendium of Protein Lysine Modifications
DRAMP	http://dramp.cpu-bioinfor.org	Antimicrobial peptides
Echinobase	https://www.echinobase.org	Echinoderm genomics
EGA	https://ega-archive.org	European Genome-Phenome Archive
GTDB	http://gtdb.ecogenomic.org	Genome Taxonomy Database
LPSN and TYGS	https://lpsn.dsmz.de, https://tygs.dsmz.de	List of Prokaryotic names with Standing in Nomenclature and Type (Strain) Genome Server
NPAtlas	https://www.npatlas.org	Natural Products Atlas
OncoSplicing	http://www.oncosplicing.com	Alternative splicing and cancer
Priority index	http://pi.well.ox.ac.uk	Drug targets for immune-mediated diseases
RGD	http://animal.nwsuaf.edu.cn/RGD	Ruminant Genome Database
SignaLink	https://slk3.netbiol.org	Tissue-specific signaling networks in model organisms
Ubibrowser	http://ubibrowser.ncpsb.org.cn/v2	Proteome-wide ubiquitin ligase/deubiquitinase-substrate interactions in eukaryotes

As usual, the Issue begins with updates from the major database providers at the European Bioinformatics Institute (EBI), the U.S. National Center for Biotechnology Information (NCBI), and the National Genomics Data Center (NGDC) in China (1–3). Thereafter, articles are placed in the usual categories: (i) nucleic acid sequence, structure and transcriptional regulation; (ii) protein sequence and structure; (iii) metabolic and signaling pathways, enzymes and networks; (iv) genomics of viruses, bacteria, protozoa and fungi; (v) genomics of human and model organisms plus comparative genomics; (vi) human genomic variation, diseases and drugs; (vii) plants and (viii) other topics, such as proteomics databases. As ever, many databases straddle multiple categories and readers are encouraged to check the full list of papers.

The COVID-19 papers include the SCoV2-MD publication (4) that is the first ‘Breakthrough’ Article in the Issue. NAR assigns Breakthrough status to papers that solve long-standing problems, or which are otherwise considered of exceptional importance. SCoV2-MD archives Molecular Dynamics simulations of all experimentally determined SARS-CoV-2 proteins. Impressively linked to phylogenetic data, it also enables users to consider the potential impact of variants on protein structure-function considering not only the usual static metrics, but also scores deriving from trajectory analysis. Elsewhere the Ensembl COVID-19 resource (5) places the SARS-CoV-2 genome in the familiar Ensembl framework, providing evolutionary insights and integrating information regarding non-coding RNA structures (from Rfam (6)) and variants. Other COVID-19 databases cover transcriptomics of infected cells, both in SCovid (7) from a single cell perspective that allows a tissue-specific view of infection and in COVID19db (8) with an emphasis on network analysis and opportunities for drug discovery. The final three databases consider the immune response to infection and the potential impact of viral genomic variants on its effectiveness. The T-cell COVID-19 Atlas (9) predicts the affinity of interaction between virus-derived peptides and HLA alleles, potentially helping to predict the susceptibility of people with different HLA genotypes to disease. Finally, ESC (10) is a compilation of SARS-CoV-2 variants with documented effects on antibody binding while VarEPS (11) considers a number of metrics, including antibody binding, in order to predict the potential impact of all possible SARS-CoV-2 variants.

In the ‘*Nucleic acid databases*’ section, several resources illustrate the trend towards single cell-level data acquisition. Two databases cover alternative polyadenylation (APA): scAPAatlas (12) offers comprehensive analysis of human and mouse data, including correlation with gene expression and links to RNA-binding proteins or miRNAs on APA-regulated regions; scAPAdb (13) extends covered species to Arabidopsis and other plants. Elsewhere scEnhancer (14) offers a single cell perspective of enhancer regions in model organisms while scMethBank (15) covers DNA methylation in human and mouse and in healthy or cancerous cells, extending the whole organism data previously captured by the same group in MethBank (16).

Following last year's flurry of databases on proteins implicated in liquid–liquid phase separation, this year sees two new resources, RNAPhaSep and RPS (17,18), capturing information on RNA molecules implicated in this phenomenon. Each curates information on experimental data and links implicated RNA molecules to information on sequence, structure, interactions, disease associations and so on. These data are hosted at popular resources including RNAInter (19) and RNALocate (20), each reporting updates this year. Transcription factors (TFs) and their binding sites are well-covered this year. The heavily used JASPAR database (21) reports a particular focus on plant TF domains as well as the introduction of word clouds as a clever visualisation of functions linked to a given TF. Factorbook (22) returns after a number of years to focus on interpretation of SNPs lying within TF-binding motifs and to facilitate downstream AI analyses with convenient Numpy format downloads. The various relationships between TFs and cell markers are described in the new database TF-Marker (23), and the same group also describe TcoFBbase (24) covering transcription cofactors and associated regulatory networks. Elsewhere, notable returning databases include MODOMICS (25) which now links to PDB structures containing modified RNA and has improved associations between RNA modification and disease; miRTarBase (26) which updates content significantly and includes new features such as editing and disease-related variants; and miRNATissueAtlas (27) which switches from microarray-based analysis to deep sequencing and expands the number of donors and tissues to give a higher resolution picture of the tissue specificity of miRNA expression.

The section on ‘*Protein sequence and structure databases*’ begins with the Issue's second ‘Breakthrough Article’. After its dramatic emergence at the most recent CASP competition (28) the AlphaFold 2 (AF2) software for protein structure prediction was quickly published (29) released open source (https://github.com/deepmind/alphafold) and applied to the complete human proteome (30). Shortly after, the AlphaFold Protein Structure Database, described here (31), was released and covers 21 proteomes. The high-quality predicted structures in the database, projected to ultimately cover UniRef90 (32) protein sequence space, provide a treasure chest of information across all aspects of biology. The impact of the database, and the software more broadly, is reflected in the incorporation of its models into cornerstone resources such as UniProt (33) and InterPro (34) but also the rapid inclusion of AF2 outputs in a number of other databases in this Issue. AF2 models and other predicted structures are now included, for example, in PDBe-KB (35) which thus graphically illustrates the complementarity between experimental structures and computational models.

Other notable new databases include the Human Proteoform Atlas (36) which assigns stable identifiers to over 37 000 proteoforms, i.e. the different protein forms that can arise combinatorially from a single gene as a result of alternative splicing, coding sequence variants and post-translational modifications. Elsewhere, the GproteinDb (37) curates a wealth of information, especially information on the selectivity of their coupling to GPCRs, for a family of great importance to therapeutic design. Among databases reporting updates is PRIDE (38) where around 500 proteomics datasets are processed each month. After processing by improved data pipelines, the results are increasingly disseminated to other key databases such as UniProt (33), Ensembl (39) and Expression Atlas (40). Other returning databases focus on proteins or protein regions lacking a single, conventionally folded structure. DisProt (41), the database for intrinsically disordered protein, reports interestingly on the nuts and bolts of curation, harnessing both professional and community biocurators in a manner supported by a refactored ontology and incentivised by the APICURON database (42). The FuzDB Update (43) reports on fuzzy interactions, i.e. those exhibiting context-dependent conformational heterogeneity, an interaction style particularly common where one or both partners are classified as intrinsically disordered. FuzDB has a new interface and expanded links out to databases covering protein structure, function and involvement in phase separation. Short linear interaction motifs are particularly common in intrinsically disordered regions and the database for such motifs in eukaryotes, ELM, contributes an Update paper (44). Among highlighted examples of newly catalogued motifs, the authors use a KEGG (45) image of endocytosis pathways to emphasise the ubiquity of motif-mediated interactions in the process and illustrate the multiple points at which diverse viruses hijack pathway components. The paper also includes an interesting window onto the variety of databases and tools used by ELM curators to sift likely real motifs from false positive matches to regular expressions.

In the ‘*Metabolic and signalling pathways*’ section, the popular Reactome database of biological processes and networks has an Update paper (46) describing an interesting collaboration with the ‘Illuminating the Druggable Genome’ (IDG) consortium (47) that helps place many ‘dark’ proteins (those that are poorly understood and/or understudied) in the context of Reactome networks. The paper also reports curation of the processes behind SARS-CoV-2 infection, a procedure interestingly expedited by first working on SAR-CoV-1 from March 2020. Reactome is one of 31 resources contributing to the molecular interaction meta-resource ConsensusPathDB which also has an Update paper (48) reporting a quadrupling in size. Options for enrichment analysis in gene set queries of the network now include regulators such as miRNA and transcription factors. Other new databases include Kincore (49), a resource that classifies protein kinase conformations and ligand types, improving our understanding of the conformational landscape of this important family and facilitating drug design. Interestingly, AlphaFold Database predictions are included and classified alongside experimental structures. Among returning databases, HMDB, the Human Metabolome Database, reports (50) a near-doubling in size, intense recuration of hundreds of the most significant metabolites, more accurately predicted spectra and improved Pathway illustrations mapping metabolites onto anatomical and (sub)-cellular features. Elsewhere, an Update paper from CAZy (51), the database of carbohydrate-active enzymes, reports significant increases in numbers of enzyme families alongside interface improvements including Krona charts (52) for taxonomic distributions of families. Finally, sister EBI resources for macromolecular interactions IntAct (53) and Complex Portal (54) each contribute an Update. IntAct has more than doubled in size since its previous publication and captures diverse information on binary molecular interactions, including a SARS-CoV-2 interactome, in particularly clean and appealing visualisations. Complex Portal, as the name suggests, focuses on stable interactions between two or more macromolecules. It has, since last publication, focused on SARS-CoV-2 and on the 300 or so complexes believed to exist in *Escherichia coli*. Ongoing work is addressing human complexes which may number around 4000.

The ‘*Microbial genomics*’ section contains Update papers from three very significant taxonomy and systematics resources most recently published elsewhere. The resources LPSN (List of Prokaryotic names with Standing in Nomenclature) and TYGS (Type Strain Genome Server) publish together (55) and describe how their colocation in 2020 facilitates data exchange and mapping between them. The paper describes the ever-increasing pace of their growth and new options for genome-scale comparison of uploaded genomes to the sequences stored in TYGS. GTDB (56) is a regularly updating genome-based taxonomy for prokaryotes which reports on a trebling of species clusters since the last publication and on possibilities to move beyond INSDC genome sequences (57) to resources such as MGnify (58) in order to better capture the full scope of metagenome-assembled genomes now available on a large scale. Several new databases focus on microbiomes and metagenomes: mBodyMap (59) helps understand the prevalence and abundance of different bacteria at different sites on the human body in health and disease; gutMGene (60) curates information on gut microbiome metabolites and human target genes with which they interact; and AMDB (61) contains gut microbe information for almost 500 animal species. Three notable databases focus on host-pathogen interactions. The well-known PHI-BASE reports (62) new pathogens and hosts, and describes the range of other databases to which it contributes annotations. The second, VEuPathDB (63), is a new name to the Issue but contains genomic and a wide variety of other information on eukaryotic pathogens, their vectors and host, information previously stored in its parent databases VectorBase (64) and EuPathDB (65), each published here. The site allows construction of sophisticated search strategies and options for analysing host-pathogen interactions are a future priority. The third, the popular VFDB (66), returns with a novel hierarchical classification of its bacterial virulence factors (VFs) into 14 categories and >100 subcategories. Chromosome maps and genomic loci can be visualised with VFs colour-coded according to their categorisation. Finally, although not focused primarily on COVID-19, two databases include it among broader information that may well help predict the appearance and spread of future viral pandemics. VThunter (67) looks at expression of viral receptors at a single-cell level across 47 animal species enabling the users to ask which species a given virus might infect or, conversely, to which viruses a given animal might be susceptible. ZOVER (68) unites and upgrades two previous databases to curate information on zoonotic viruses carried by rodent, bat and insect vectors: information includes mapping of viral families to host species and geographical virus distributions.

In the next section (‘*Genomics of human and model organisms plus comparative genomics*’) a number of important databases contribute updates. Ensembl reports (69) on addressing the ever-increasing influx of data with new, more efficient workflows and a new Rapid Release platform which together allowed more than 200 genomes to be covered in around a year. A new interface is being implemented after researching user interaction patterns, and non-vertebrate genomes are also included for the first time as the database continues on the path to merger with Ensembl genomes. The paper on the latter (70) reports the largest content increase yet seen including almost 500 new fungal genomes. Other interesting developments include proteome-based removal of redundancy in hosted bacterial genomes, a move to better support pangenomes and inclusion of AlphaFold models for Arabidopsis. The USCS Genome Browser Update paper (71) describes a variety of new assemblies, tracks and display features, including support for different fonts in the genome browser display. There is also a clever SARS-CoV-2 feature allowing placement of a new genome in phylogenetic context, facilitating comparisons between sequences and with annotation tracks.

Elsewhere, a number of comparative genomics resources focusing on species of biological or agricultural importance feature. The Ruminant Genome Database (72) paper reports significant expansion of its multi-omics content throughout. Insects are the focus of three returning database: InsectBase (73) reports dramatic increases in content as well as new features focusing on ncRNA–mRNA interactions and likely horizontal gene transfer; Hymenoptera Genome Database (74) covers a tripling of covered species and a focus on better Gene Ontology (75) assignments allowing, for example, better on-site GO enrichment analysis; and FlyAtlas 2 (76) enhances its (sub-) tissue-specific gene expression data and introduces a new co-expression tool. As usual, aspects of human genomics feature strongly. The new PopHumanVar database (77) builds on previous work (78,79), calculating and assembling information on variants, in order to help identify those responsible for selective sweeps. 3DSNP (80), continues its work in contextualising variants using information on 3D chromosome conformation, now expanding to cover structural variation such as inversions, deletions, duplications, and insertions. A new database SomaMutDB (81) covers mutations—SNVs and small insertions or deletions—in somatic cells, linking them to data such as regulatory elements and gene expression data, to facilitate their analysis and comparison with much more common cancer-related mutation data. The publication from the European Genome-Phenome Archive (82), with its potentially identifiable genetic, phenotypic and clinical human data, coincides with an alteration to the guidelines for acceptance into the Database Issue (available online at https://academic.oup.com/nar/pages/ms_prep_database). Previously, the Issue blanket disallowed any form of registration: henceforth such registration is allowed, but only in specific cases where it is legally required in order to protect the integrity of potentially identifiable human data. The EGA paper includes a detailed discussion of its access and download protocols, and of prospects for future sharing of such data.

The section on ‘*Human genomic variation, diseases and drugs*’ contains papers on two new resources for linking genetic variation to disease. VannoPortal (83) integrates no fewer than 40 data sources to provide impressively comprehensive linkages between variants and diseases or traits, and boasts a particularly clean and responsive interface. ConVarT (84) takes the approach of mapping equivalent variants between orthologous protein pairs between human and model organisms such as *Caenorhabditis elegans*. This allows experimental data on variant pathogenicity obtained from model organisms to help interpret the consequences of human variants. Molecules of the immune system are the focus of both the venerable IMGT^®^ databases which contributes an update (85), and the new human Antigen Receptor database (huARdb (86)) which exploits new single-cell immune profiling and transcriptomics to reveal individual clonotypes of T-cell and B-cell receptors (TCRs and BCRs). Notably, huARdb offers stable URLs for results of analyses of user data at the site to facilitate interactive data sharing. Two further databases deal with antibodies, including nanobodies - antibodies consisting of a single monomeric variable domain. INDI (87) collects sequences and structures plus associated metadata from a variety of sources and allows various modes of sequence or text search. The authors envisage the dataset being valuable for computational efforts towards nanobody design. SAbDab focuses on antibody structures, updated weekly, and here describes increases in content along with a new SAbDab-nano section dedicated to nanobodies (88).

Elsewhere, drug combinations and interactions are covered by two new databases. DDInter (89) mines the literature for information on drug–drug interactions, classifying the results (synergy, antagonism etc.) and presenting interactions in a variety of attractive visualisations. NPCDR (90) works in a similar area but focuses on cases where at least one of the drugs involved is based on a natural product. Cellular responses to drugs are captured by the new CeDR database (91), which uses single cell transcriptomics data to capture the characteristic drug responses of different cells and tissues, in human and mouse and in health and disease. In a similar area, CTR-DB (92) contains clinical transcriptomics data from cancer patients, both pre-treatment and drug-induced. A myriad of analytical options maximise the data's value in, for example, biomarker discovery and understanding drug resistance mechanisms. Other new cancer-related databases include CancerMIRNome (93) that covers miRNAs in cancer cells and offers particularly rich analytical options; CancerSCEM (94) that offers similarly diverse options for studying single cancer cell gene expression data; GPEdit (95) which links A-to-I RNA editing in cancer cells to pharmacogenomic responses and patient survival; and OncoDB (96), which focuses on the contributions of gene expression dysregulation and viral infection to cancer development and progression. This year also sees Update papers from two major general resources in drug design. The IUPHAR/BPS guide to PHARMACOLOGY (97) reports on its efforts to curate information on drugs and drug targets for SARS-CoV-2, as well as updates to its sections on Malaria and antibacterials. The paper from the Therapeutic Target Database (TTD) (98) reports significant updates including many new kinds of data including information on weak or non-binders of targets, prodrug-drug pairs and AlphaFold models of drug targets for which experimental structures are not yet available. Finally, it's a pleasure to welcome the European Variation Archive (EVA) (99) to the Issue, a full eight years after its genesis. In that time its content has grown dramatically to now cover over 3 billion variants.

The ‘*Plant database*’ section includes an Update paper from the popular comparative genomics resource PLAZA (100) which reports a near-doubling of species covered and new and improved features throughout, including the API. The paper on BRAD (101), the dedicated Brassica database, reports a particular focus on synteny analysis tools and looks forward to accommodating the more diverse omics data and pangenome information now becoming available for the Family. Plant ncRNA is covered by returning databases GreeNC (102), with its focus on lncRNA, and PmiREN (103) which doubles its content of miRNA entries. The latter offers an impressive array of new features for functional and evolutionary exploration including gene regulatory elements, target annotations, variants and phylogenetic trees. Finally, welcome new arrivals include PlantGSAD (104) which provides >200 000 gene sets across 44 families, sets based on a notably diverse set of properties; and qPTMplants (105) which curates data, including quantitative information, on post-translational modifications (PTM) across 43 species. The latter features an interesting discussion of PTM crosstalk identified in the database.

The final ‘*Other databases*’ section includes Update papers from major proteomics resources. iProX, a member of the ProteomeXchange consortium (106) as now processed almost 100 TB of submitted data and reports new features such as an efficient reanalysis platform and an API (107). ProteomicsDB also reports a new API, generated with reference to FAIR principles (108), alongside a new interface with fresh visualisation options (109). An update from Proteome-p*I* (110) reports on a more than trebling of its content of predicted p*I* (isoelectric point) and p*K*_a_ values for proteins and *in silico* digested peptides, parameters relevant to proteomics and other biophysical experiments. Finally, two new databases curate information previously only inconveniently scattered through the literature. dNTPpoolDB contains concentrations of deoxyribonucleotide triphosphates in different species, cells and experimental conditions (111) while ProNAB contains >20 000 data points on binding affinity of proteins (wild-type and mutant) for DNA or RNA (112).

## NAR ONLINE MOLECULAR BIOLOGY DATABASE COLLECTION

We are pleased to include 1645 entries in this 29th release of the NAR online Molecular Database Collection (available at http://www.oxfordjournals.org/nar/database/c/). We have updated 317 entries, 89 new resources were added and 80 entries were removed in our ongoing effort to provide an up-to-date collection. We encourage authors to send their updates (in plain text according to the template found in http://www.oxfordjournals.org/nar/database/summary/1) to xose.m.fernandez@gmail.com.
